# Emergence of co-expression in gene regulatory networks

**DOI:** 10.1371/journal.pone.0247671

**Published:** 2021-04-01

**Authors:** Wencheng Yin, Luis Mendoza, Jimena Monzon-Sandoval, Araxi O. Urrutia, Humberto Gutierrez

**Affiliations:** 1 Centre for Computational Biology, University of Birmingham, Birmingham, United Kingdom; 2 Instituto de Investigaciones Biomédicas, UNAM, Ciudad de México, México; 3 UK Dementia Research Institute at Cardiff University, Cardiff, United Kingdom; 4 Instituto de Ecología, UNAM, Ciudad de México, México; 5 Department of Biology and Biochemistry, Milner Centre for Evolution, University of Bath, Bath, United Kingdom; 6 Instituto Nacional de Medicina Genómica, Ciudad de México, México; Universita degli Studi di Torino, ITALY

## Abstract

Transcriptomes are known to organize themselves into gene co-expression clusters or modules where groups of genes display distinct patterns of coordinated or synchronous expression across independent biological samples. The functional significance of these co-expression clusters is suggested by the fact that highly coexpressed groups of genes tend to be enriched in genes involved in common functions and biological processes. While gene co-expression is widely assumed to reflect close regulatory proximity, the validity of this assumption remains unclear. Here we use a simple synthetic gene regulatory network (GRN) model and contrast the resulting co-expression structure produced by these networks with their known regulatory architecture and with the co-expression structure measured in available human expression data. Using randomization tests, we found that the levels of co-expression observed in simulated expression data were, just as with empirical data, significantly higher than expected by chance. When examining the source of correlated expression, we found that individual regulators, both in simulated and experimental data, fail, on average, to display correlated expression with their immediate targets. However, highly correlated gene pairs tend to share at least one common regulator, while most gene pairs sharing common regulators do not necessarily display correlated expression. Our results demonstrate that widespread co-expression naturally emerges in regulatory networks, and that it is a reliable and direct indicator of active co-regulation in a given cellular context.

## Introduction

Organismal development arises from the interplay of thousands of gene products governed by an underlying network of regulatory interactions [1, 2]. Understanding the detailed architecture of these gene regulatory networks (GRNs) is an important interdisciplinary challenge for both molecular genetics and developmental biology [3–5]. While gene expression can respond to changes in numerous environmental variables, the dynamic expression of each gene is fully driven by the underlying structure of the genetic regulatory architecture (Bodaker, et al., 2013; Das, et al., 2004; Schlitt and Brazma, 2007) [6–8]. Because genes exert a continuous, direct or indirect, regulatory influence on each other, the collective profile of gene expression in a given cell type or tissue is never a static cellular feature (i.e., a constant level of expression for each or most genes), even under extremely stable physiological conditions (Schlitt and Brazma, 2007) [8]. Instead, it is the dynamic equilibrium reached by the whole genetic network what will determine the functional competence of a given cell or tissue under defined physiological conditions (Hartwell, et al., 1999; Payne and Wagner, 2013; Payne and Wagner, 2015; Stead, et al., 2006; Sterner, et al., 2012) [1, 2, 9–11]. One way of capturing the nature of this dynamic equilibrium states is by examining the collective pattern of gene expression variations in a given cell type or tissue.

Along these lines, transcriptomes are well-known to organize themselves into gene co-expression clusters or modules where groups of genes display distinct patterns of coordinated or synchronous expression across independent biological samples [12–18]. The functional significance of these co-expression clusters is suggested by the fact that highly coexpressed groups of genes tend to be enriched in genes involved in common functions and biological processes [13, 19, 20]. While this co-expresion structure is believed to reflect, in some sense, the underlying regulatory architecture of the genetic machinery, the exact source of these coordinated patterns of expression remains unclear. More specifically, co-expression is commonly assumed to be the result of genes sharing common regulators (i.e., jointly targeted by shared transcription factors), however this assumption has never been formally tested as correlated expression could also emerge in principle even in the absence of regulatory proximity.

Previous attempts to assess whether genes displaying high expression correlation are more likely to share transcription factor binding sites (TFBS), when compared to those with low expression correlation, have been carried out with conflicting results. Thus, for instance, in the single-cell model Saccharomyces cerevisiae, for which both extensive microarray expression data and experimentally verified TFBS data exist [12, 17, 21], only gene pairs with a very high degree of co-expression share significantly larger number of TFBS [12]. However, a similar analysis carried out in Drosophila, a much more complex multicellular model, found that gene pairs with high expression correlations do not share significantly larger numbers of TFBS [13].

Understanding the relationship between the dynamics of expression profiles, and the underlying regulatory architecture, will eventually allow us to develop adequate tools to better interpret the effect of regulatory relationships under both normal and pathological conditions. At its most basic level, GRNs can be represented as a web of transcription factor proteins binding specific regulatory sequences on target genes to control their spatial and temporal expression [5, 8, 22–26]. This means that actual genetic networks integrate vast webs comprised of thousands of genes interlinked by either positive or negative regulatory interactions depending on whether a given transcriptional regulator exerts a positive or negative influence on the rate of transcription of targeted genes [8, 27, 28]. Synthetic computational models of GRNs offer an adequate and experimentally tractable tool to investigate the relationship between the gene regulatory architectures on the one hand and the expected dynamical patterns of gene expression on the other [27–29]. However, the objective of the present study is not to present a method for regulatory network inference. While synthetic gene regulatory network models have been extensively used as benchmarks for regulatory network inference methods [27–29], no previous study has actually used them to explore the potential source of co-expression itself.

In this study, we combine brain-derived gene expression data with synthetic GRN model simulations to investigate: first, if the correlated structure observed in natural transcriptomes deviates in any way from random expectations. Second, where these correlations could actually come from; and, third, the extent to which co-expressed pairs of genes are expected to be in close regulatory proximity compared to random background gene pairs. This approach will allow us to gain mechanistic insights into how co-expression patterns relate to the regulatory interactions between genes, and will provide a theoretical framework to properly interpret co-expression dynamics in living cells both in health and disease.

## Methods

### The GRN model

For this study, we used a simple GRN model aimed at capturing the essential statistical features of real transcriptional regulatory networks: The regulatory architecture of our synthetic GRN was represented by an interaction network composed of a defined number of nodes and arrows, where each node represents a gene, and arrows linking them represent regulatory interactions. We specifically used networks consisting on 1000 nodes, each one regulated by a variable number of nodes randomly drawn from the entire network, with a set minimum number of regulators per gene. As detailed analysis of promoter architecture (transcription factor binding site distribution) for the human genome has revealed a distribution of regulators to a target gene closely fitting a power law [30], the number of regulators *k* per gene i was set to follow a power law distribution defined as:

$$k_{i}=min+round\left(1/x^{1⁄2}\right)$$ (1)

Where min is the minimum number of regulators and x is a uniformly distributed random number. With the second term rounded, as k_i_ is set to be a whole number.

The expression state of a gene is represented by a real number and the initial state of the network was set to be a random number for each gene/node drawn from a uniform distribution between 0 and 100. The rate of expression of a gene under the influence of a single regulator is given by a sigmoid function of the level of expression of that single regulator.

$$f_{i}=A\left(\frac{1}{{1+e}^{B-{CG}_{i}}}\right)+1$$ (2)

Where G_i_ is the level of expression of the regulator; A, B and C are parameters controlling the maximum value, the inflexion point (threshold) and slope at that point for this function (S1 File). Note that f_i_ is defined in the interval [1, A+1] (where the minimum influence exerted by G_i_ is 1 and the maximum is A+1).

This model assumes that the expression level of a target gene (under the influence of a single regulator), is itself the rate f with which that regulator will contribute to the expression of the target, and that when several regulators act on a gene, they interact cooperatively.

If f_i_ is the rate of expression induced by the regulator *i*, the overall expression of the target (*Ex*) under the influence of *k* regulators will be given by:

$$Ex=f_{1}*f_{2}*f_{3}*\ldots f_{k}$$ (3)

The expression state of each gene was simulated over a succession for 500 discrete time points, with the state of a given node at time t being a function of the state of its regulators at time t-1. Parameters associated to the sigmoid function used in the model were tuned to avoid frozen states (that is, where all nodes become locked in constant values, S1A Fig and S1 File). This model, was implemented in Matlab (R2017b) and for every simulation, expression profile time courses were stored as CSV files for further analysis (the corresponding Matlab script used in this study can be found in S1 File). Regulatory interaction networks were stored as edge list matrices for further analysis (an example of a synthetic expression dataset and the associated network edge list can be found in S2 and S3 Files).

### Source and processing of empirical expression data

RNA-seq human brain expression data (RPKM values) was sourced from the BrainSpan database (http://www.brainspan.org), corresponding to a total of 18 separate brain regions from a range of ages going from eight weeks post-conception through to 40 years of age. CAGE expression data for 60 brain tissue samples from the FANTOM5 dataset were obtained from Hurst et al., 2014 [30]. Quantile normalized expression values from both datasets were annotated to Ensembl gene IDs (for Brain span data, individual expression values for more than one Ensembl transcript ID annotated to the same Ensembl gene ID were averaged). Genes displaying a standard deviation = 0.0 were excluded.

Annotations of Entrez IDs for transcription factors MYC, PAX2 and SRY target sites were obtained from the Molecular Signatures Database v4.0 (MSigDB; http://www.broadinstitute.org/gsea/msigdb/index.jsp). These annotations are based on transcription factor (TF) binding sites compiled in the TRANSFAC (version 7.4, http://www.gene-regulation.com/) database. Entrez IDs and gene symbols were mapped to Ensembl IDs with a correspondence table downloaded from Ensembl’s Biomart (S4 and S5 Files).

### Co-expression network analysis

Weighted gene co-expression network analysis was carried out based on pairwise correlations between the quantile-normalized expression profiles obtained from either the BrainSpan database (http://www.brainspan.org) or the FANTOM5 dataset [30] for over 21 000 genes. Spearman correlations were used here in order not to assume linear relationships between coexpressed genes [18], and genes displaying standard deviation equal to zero, if any, where eliminated in both empirical and simulated data Unsupervised hierarchical clustering was used to detect groups, or modules, of highly coexpressed genes following the method described by Zhang & Horvath, 2005 [18].

### Topological distance analysis

Using Matlab’s network analysis functions, and based on the connectivity network of each simulated GRN, we measured the topological distance between pairs of highly correlated genes. Topological distance is defined as the minimum number of nodes linking any two nodes in a directed or undirected network. In this study we used undirected topological distance between correlated gene pairs. These measurements were compared to the average topological distances between randomly paired genes sampled from the same network and this comparison was statistically tested for significant differences using a student’s t test.

### Programming and statistical software

All additional large-scale calculations, numerical simulations and statistical analyses were carried out in R.

## Results

We started by directly examining the correlated structure observed in empirical expression data and whether it deviates in any way from random expectations.

**Fig 1:**
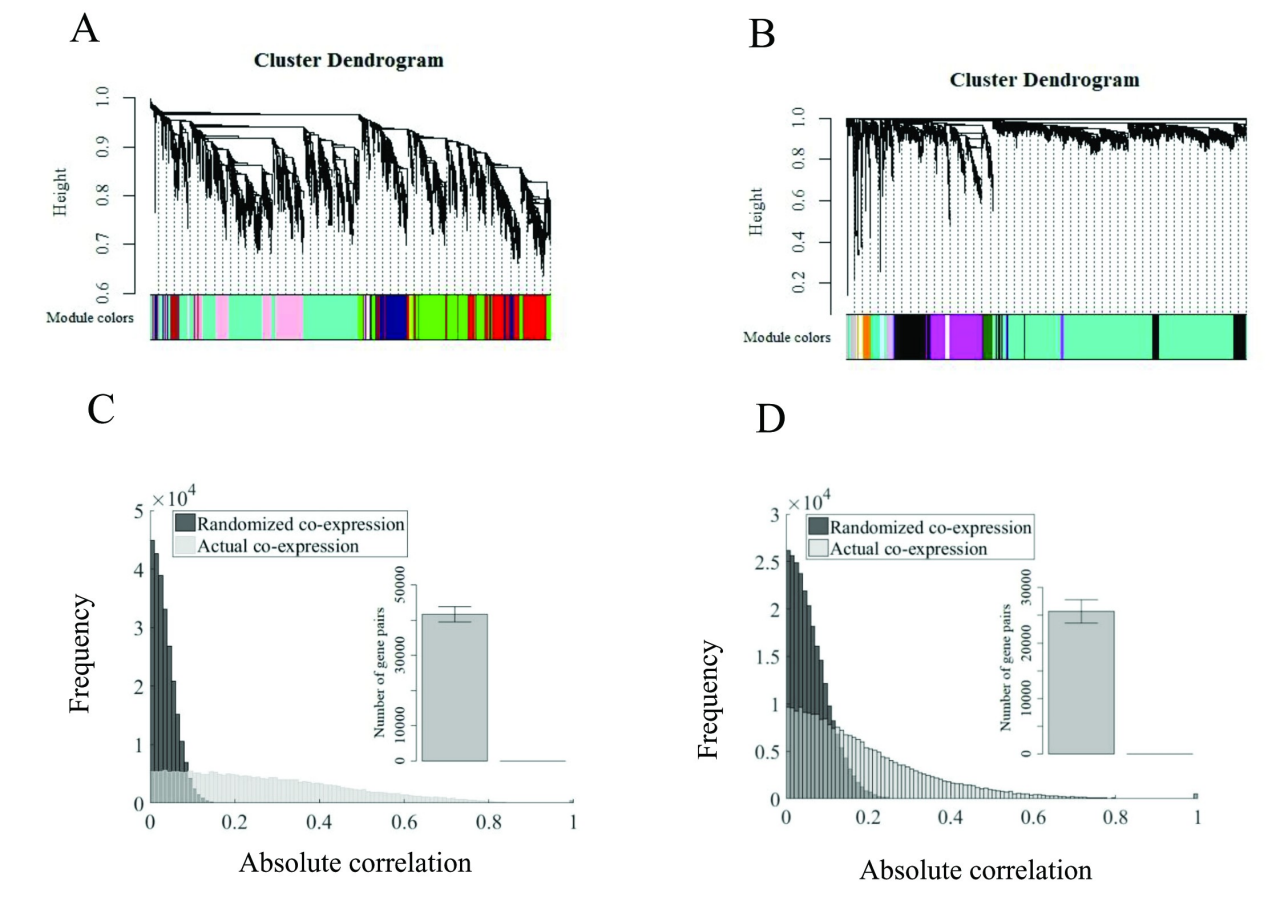
Frequency of co-expression is higher than expected by chance in natural transcriptomes. **A** and B) Typical co-expression structure and modular organiza-tion of a random sample of 1000 genes from the human brain transcriptome (Brain-span dataset) and 60 brain tissue samples obtained from the Fantom5 dataset. C) and D) Distribution of absolute correlations of all gene pairs in a random sample of 1000 genes compared with the distribution resulting from random permutations of expression data in the same genes. Inset: Bars show the mean (±SEM) number of highly correlated pairs (/R/>0.5) among 1000 independent samples of 1000 genes each compared with the expected mean number of highly correlated pairs (absolute correlation) resulting from random permutations of the gene expression values in the same samples.

To this end, we used available human expression data derived from two separate datasets (See methods) and calculated the correlation coefficient of all possible pairs of genes. Cluster analysis based on co-expression reveals a clear structure in the correlated patterns of the entire transcriptome. Fig 1A and 1B, show the clustered structure of two representative random samples of 1000 genes. As correlated pairs of genes are likely to occur by mere chance among millions of possible gene pairs, we next confirmed that the frequency with which genes display highly correlated expression actually exceeds chance expectations. To this end, we examined the global distribution of absolute correlation values and compared it with the expected distribution of correlation coefficients resulting from randomly permuting each gene’s expression values across all the included samples (see methods). As shown in Fig 1C and 1D, the empirical distribution of correlation values shows a clear spread of large correlations when compared with the distribution resulting from the randomized data. The number of correlated gene pairs with a coefficient /R/ > 0.5 was also significantly larger relative to chance expectations, confirming that the highly correlated structure observed in transcriptomes is not the result of expected random statistical associations (insets in Fig 1C and 1D).

In order to identify the possible origin of excessive correlations in GRNs, we generated a simple computational gene network model of regulatory interactions capable of simulating expression dynamics based on simplified but realistic kinetic properties (see methods section and S1 Fig). The model generates random networks representing regulatory interactions between genes, according to defined statistical topologies. Direct interactions between individual regulators and their targets are modelled by a simple sigmoid function dependent on two main parameters: the inflexion point (threshold) and the saturating value (the maximum target expression in response to the regulator’s expression; see methods). Under a wide range of values for these parameters, successive iterations of this model generate fluid and dynamic expression time series that can be easily tuned to avoid frozen states (i.e., where all nodes become locked in constant values, S1 Fig and S1 File). Starting from any arbitrary initial set of expression values the dynamics of this model quickly reaches realistic equilibrium distributions of gene expression readily comparable with those observed in natural transcriptomes, based on frequency of transcript read counts (S1B–S1D Fig).

**Fig 2:**
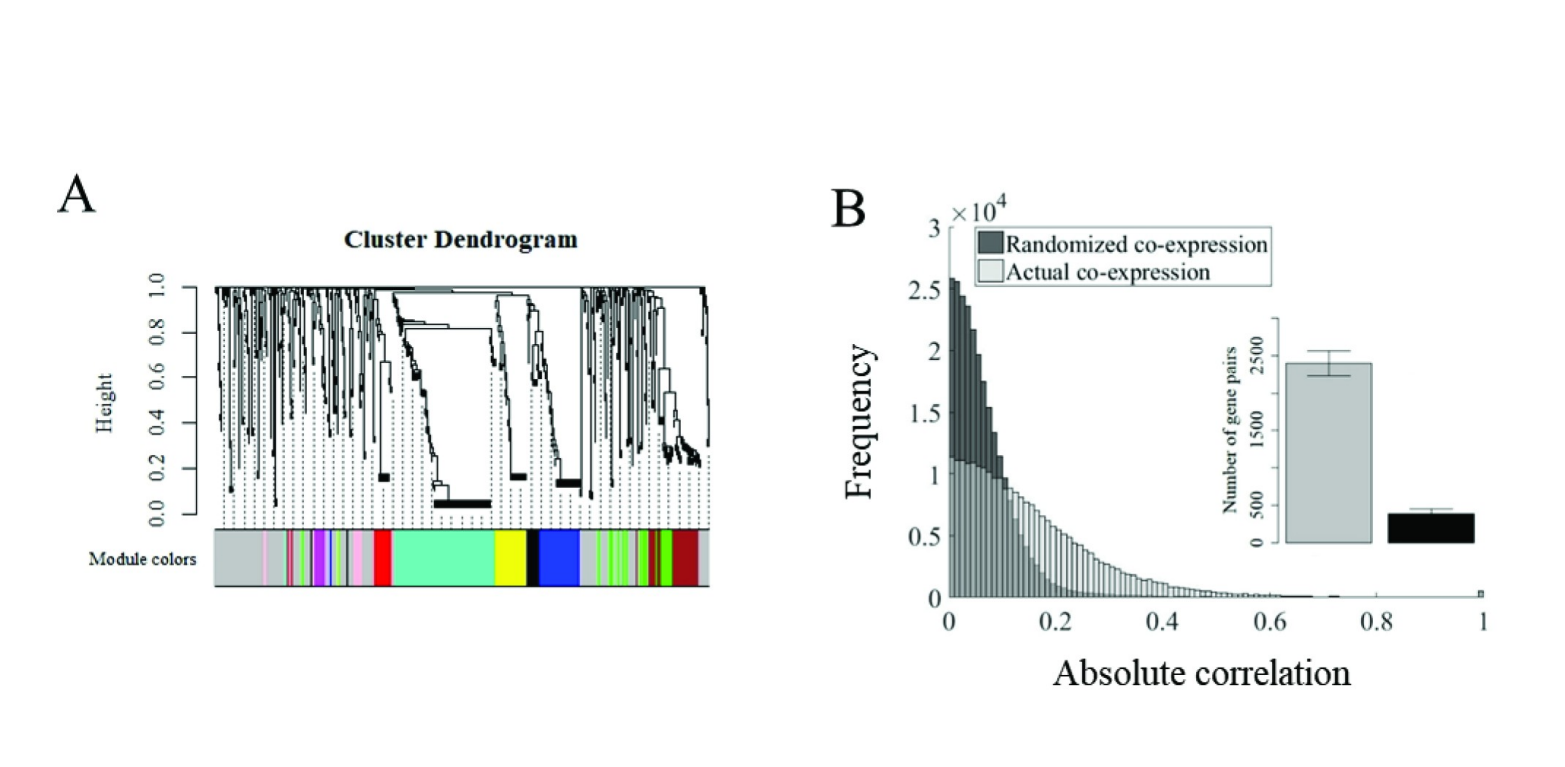
High co-expression naturally emerge in synthetic gene regulatory networks. A) Co-expression clustering dendrogram based on expression data generat-ed by a synthetic GRN after 1000 time steps (iterations). B) The distribution of the absolute correlation of all synthetic gene pairs in in a simulated network of 1000 genes compared with the distribution resulting from random permutations of the same expression data. Inset: Bars show the mean (±SEM) number of highly corre-lated pairs (/R/>0.5) obtained from 1000 independent GRN simulations compared with the expected mean number of highly correlated pairs (absolute correlation) resulting from random permutations of the gene expression values of the same networks.

Using this model, we simulated expression data in networks of 1000 nodes for 500 time points (iterations) and generated the corresponding correlation matrix for all possible gene pairs in the network. Cluster analysis based on co-expression reveals a clear clustered structure, indicating that transcriptomes generated by the synthetic model organize themselves into co-expression modules (Fig 2A).

We then asked if the frequency of high correlations, in these synthetic GRNs, also exceeds chance expectations. Using the approach we followed earlier, we obtained the global distribution of absolute correlation values and compared it with the expected distribution of correlation coefficients resulting from randomly permuting each gene’s expression across all samples. As shown in Fig 2 the distribution of absolute correlation values in synthetic networks shows again a clear spread of large correlations in stark contrast with the distribution resulting from the randomized data (Fig 2B). The number of correlated gene pairs with /R/ > 0.5 was also significantly larger relative to chance expectations (χ2 = 1969, p<10^−16^, inset Fig 2B). These results demonstrate that the distinct co-expression structure observed in natural transcriptomes also emerges in the dynamics of synthetic regulatory networks. While the co-expression structure observed in Fig 2, was based on a network architecture consisting of a power law distribution of regulators per target (also used in all following analyses), we also explored alternative architectures (fixed number of regulator and a normal distribution of regulators per target, respectively) where a similar excess of co-expression emerges (S2 Fig). These results offer the opportunity to investigate the source of highly correlated behaviour in regulatory networks in general and in the genetic regulatory networks in particular.

**Fig 3:**
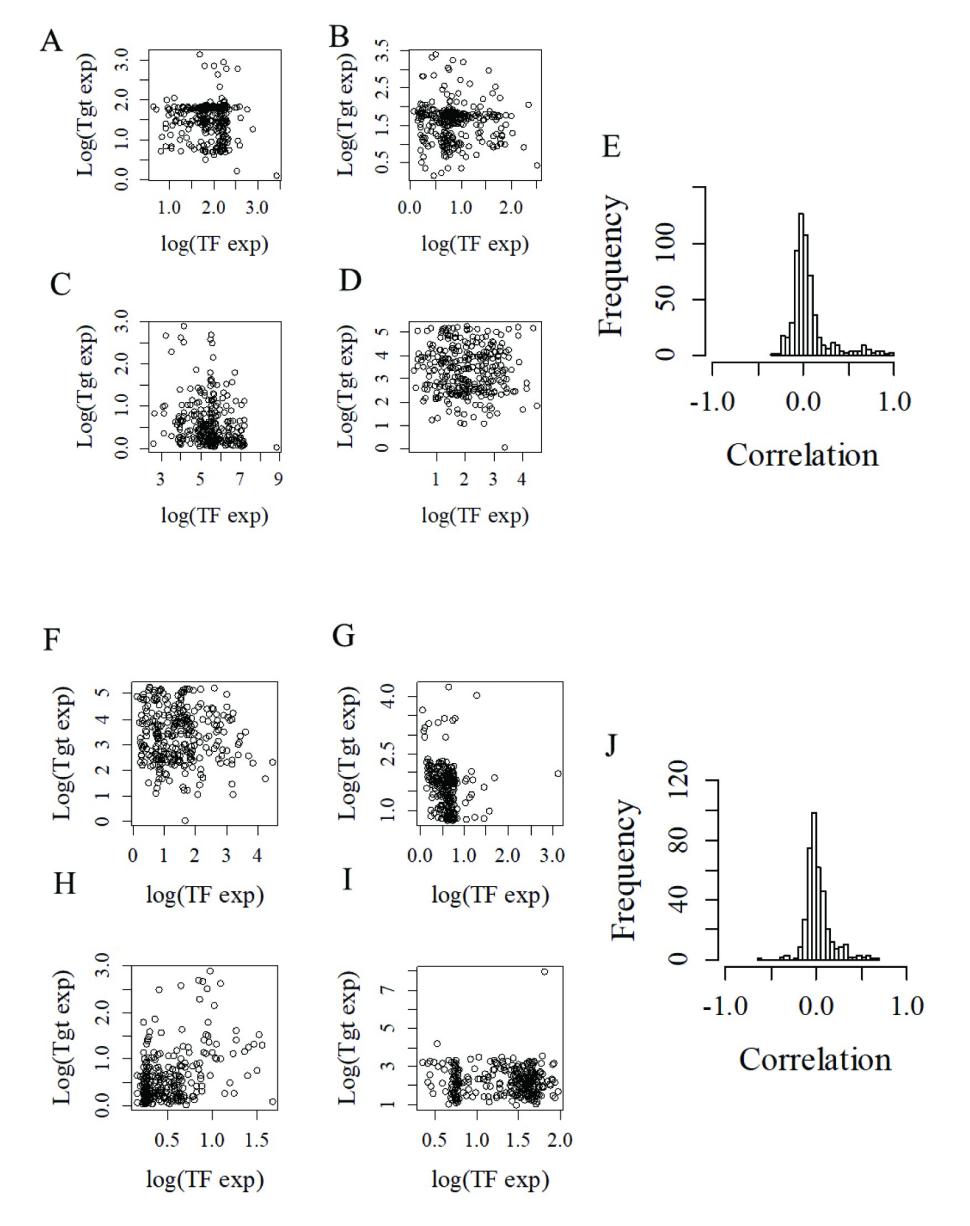
Regulators and direct targets show poorly correlated expression levels in synthetic GRNs. A-D) Typical scatter graphs showing the level of expression of four independent target genes (Tgt, expressed as log-transformed values) as a function of the level of expression of one direct positive regulator (TF). E) Distribution of correlation values of all individual regulator-target pairs involving only positive regulators. F-I) Typical scatter graphs showing the level of expression of four independent target genes as a function of the level of expression of one direct negative regulator (TF). J) Distribution of correlation values of all individual regulator-target pairs involving only negative regulators.

Along these lines, we asked if the simplest possible regulatory interaction between any two genes (i.e. an individual regulator and its direct target or targets) results in a correlated behavior at the level of expression. In principle, we would expect direct regulatory interactions to result in strong correlations, either positive or negative for positive or negative regulators respectively. To test this assumption, we identified in our synthetic GRNs all individual gene pairs linked by a direct regulatory interaction (regulator-target pairs), and examined their association. Fig 3A–3D, shows scatter plots for four randomly chosen examples of individual positive regulator-target pairs displaying the observed level of expression of each of these targets as a function of the regulator’s expression. These examples suggest a clear lack of association between the level of expression of individual targets and their direct positive regulators. In order to confirm this observation we calculated the correlation coefficient of all existing gene pairs linked by a positive regulatory interaction in the network (>3000 regulator-target pairs). As shown in Fig 3E, positive regulators and targets display, on average, an extremely poor or no correlated expression. This result was replicated when only negative regulators where considered (Fig 3F–3J), further confirming a general lack of correlated expression between regulators and their direct targets. When these same analyses were carried out using alternative architectures (fixed number of regulator and a normal distribution of regulators per target), a similar lack of average correlated expression between regulators (both positive and negative) and targets (see S3 Fig) was observed. These results imply that correlated expression does not emerge as the result of the direct interaction between a regulator’s gene (i.e., transcription factor), and their immediately targeted genes.

**Fig 4:**
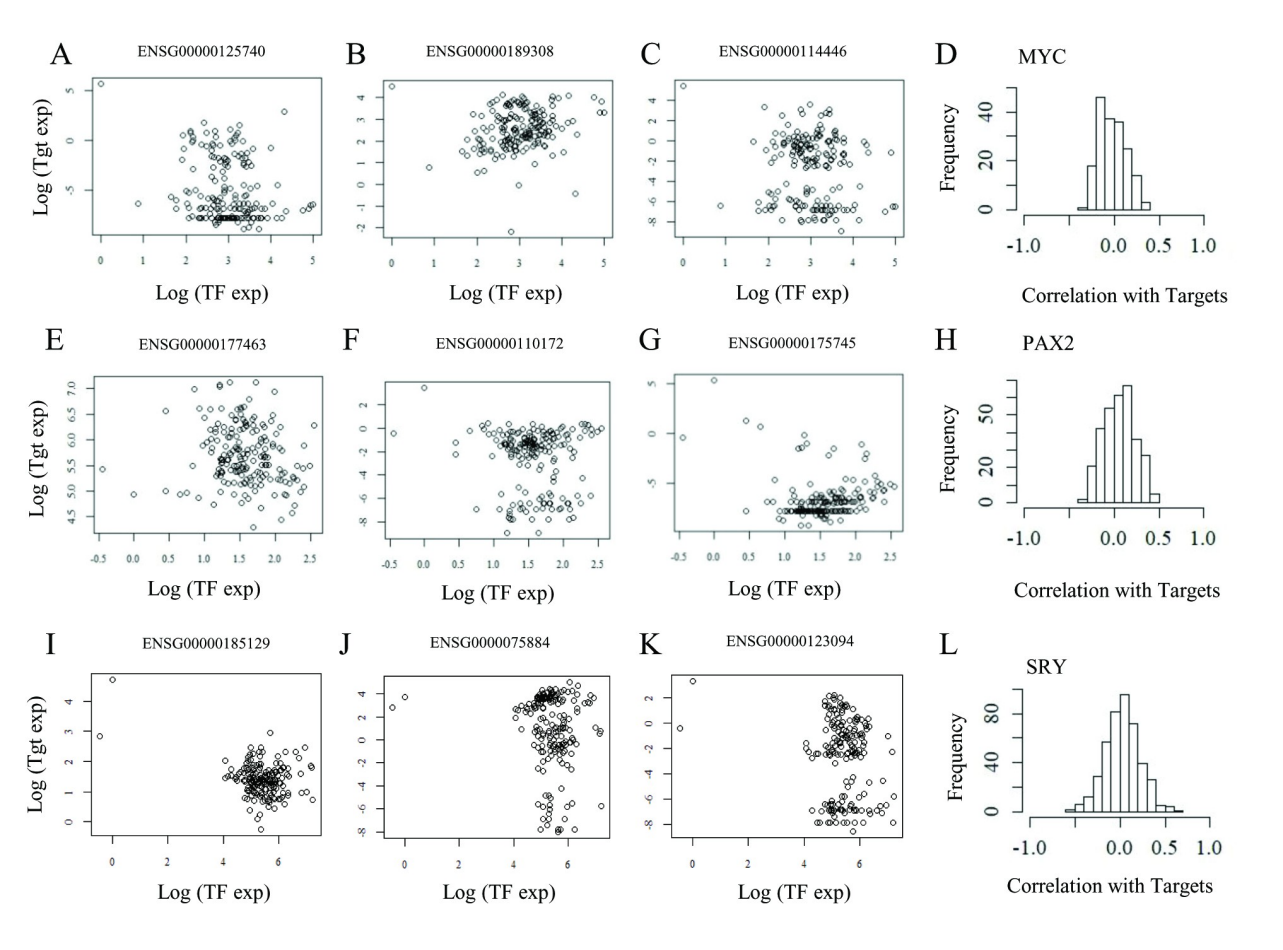
Transcriptional regulators in natural transcriptomes are poorly correlated with their individual targets. (A-C): Scatter graphs showing the level of expression of three out of 400 known targets (Tgt, expressed as log-transformed values) of the transcription factor MYC, as a function of the level of expression of this same transcription factor (TF). D) Distribution of correlations between MYC expression against 400 identified MYC targets. (E-G): Scatter graphs showing the level of expression of three independent targets of the transcription factor PAX2, as a function of the level of expression of this same transcription factor. H) Distribution of correlations between PAX2 expression against 400 identified targets. (I-K): Scatter graphs showing the level of expression of three independent targets of the transcription factor SRY, as a function of the level of expression of this same transcription factor. L) Distribution of correlations between SRY expression against 400 identified targets. Ensembl IDs for each individual target in the scatter graphs are indicated. For each transcription factor, the scatter graph shown correspond to percentiles 25, 50 and 75 of the correlation distribution for each set of targets.

To test if the same lack of correlation between regulators and targets is found between gene regulators (transcription factors) and their direct target genes in experimental data, we examined expression data of three representative and well-known transcription factors, MYC, PAX2 and SRY, and their direct known targets. We identified over 400 direct target genes for each of these three transcription factors using the MsigDB dataset, which compiles data for known transcription factor binding sites across all annotated human genes (see methods). Using normalized expression data from the Brainspan dataset, we calculated the correlation between these transcription factors and each of their targets. Fig 4A–4D, shows a poor average correlated expression between MYC and its targets. This uncorrelated behaviour was also observed for PAX2 (Fig 4E–4H) and SRY (Fig 4I–4L). It is worth noting that these distributions are much narrower than those resulting from equally-sized samples of random gene pairs drawn from the background gene population. These results show that the expression of these three representative and well-known transcription factors, is poorly correlated with the expression of their known direct targets.

**Fig 5:**
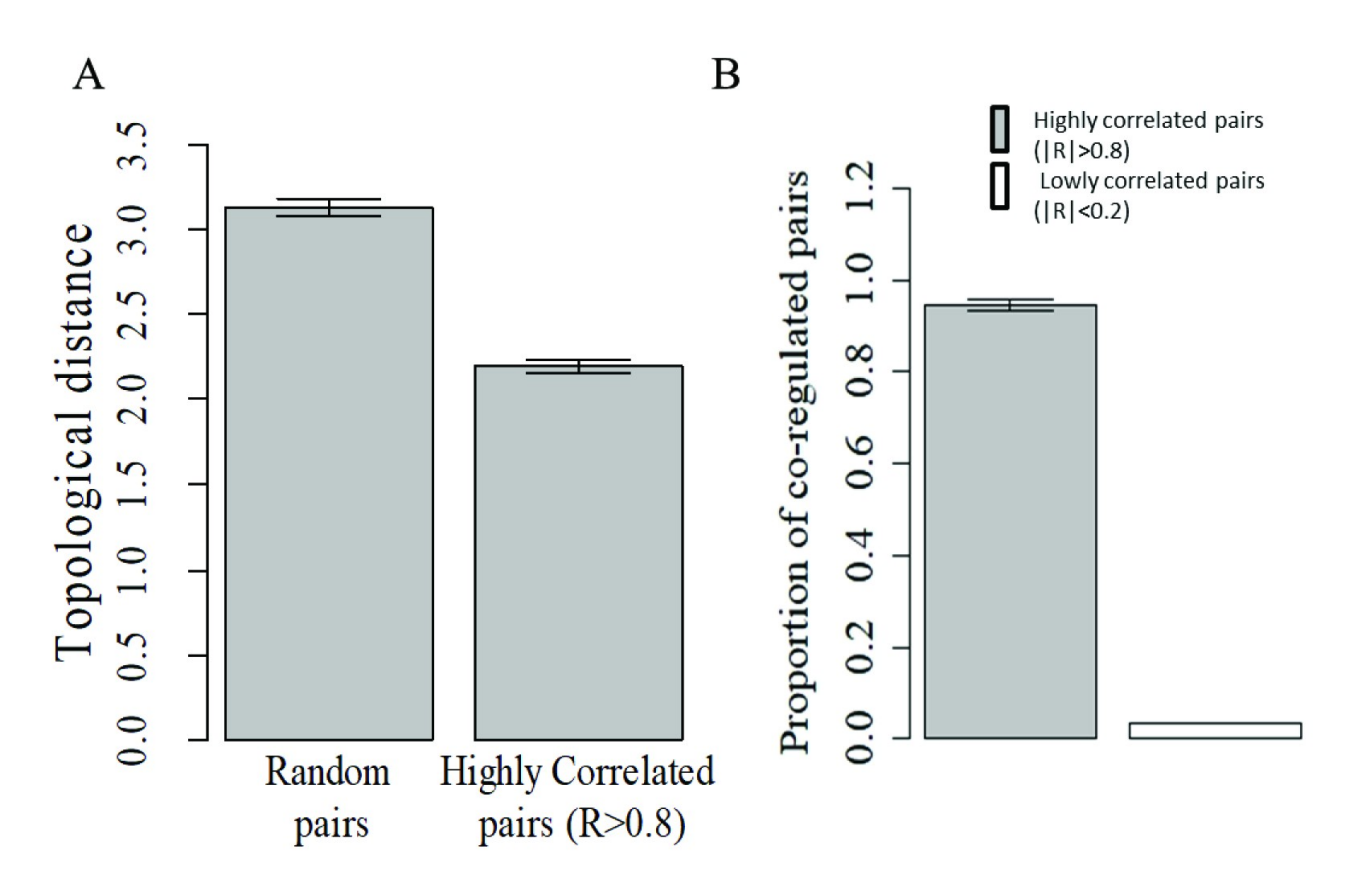
Correlated pairs of genes share common regulators in synthetic GRN. A) Typical average topological distance (a measure of regulatory proximity in the GRN) between pairs of highly correlated genes (/R/ > 0.8) compared with the average distance between random pairs of background genes in the synthetic regulatory network. In both cases, the associated p value in a T test was below 10–16. B) Bar chart showing the average proportion (±S.E.M) of pairs of genes sharing a common regulator among either highly correlated pairs (/R/ >0.8) or lowly correlated pairs (/R/ < 0.2) found in 1000 independent simulations.

Because a transcription factor-target pair is the simplest possible regulatory interaction between any two genes, the observed general lack of co-expression between regulators and their targets suggests that correlated expression in GRNs can only be the result of more indirect associations. In order to assess the regulatory proximity of correlated genes in a GRN, we measured the undirected topological distance (see methods) between the top most highly correlated gene pairs (/R/ > 0.8) in our synthetic GRNs, and compared this measure with the average topological distance between random background gene pairs. As shows in Fig 5A the average topological distance between highly correlated gene pairs in the regulatory network was significantly shorter than that observed between random background genes (p < 10^−13^, student T test). Crucially, the average distance between highly correlated gene pairs was very close to two, regardless of the topology or density of regulatory interactions in the network, strongly suggesting that highly correlated genes tend to be linked by only one regulatory component. There are only three possible configurations involving a directed network linking any two focus genes through a third one. However and in the context of co-expression, only one configurations is likely to lead to correlated expression of two genes if they need to be linked by a third one: two genes under the control of a common regulator. We tested this hypothesis by measuring, in our simulated GRNs, the proportion of highly correlated gene pairs (/R/ >0.8) sharing a common regulator compared to the proportion of lowly correlated pairs (/R/ <0.2) also sharing a common regulator. As shown in Fig 5B, the vast majority of highly correlated gene pairs (>90%) shared common regulators across 1000 independent simulations of GRNs. By contrast, only an extremely low proportion of lowly correlated gene pairs shared common regulators (p <10^−16^, Student T test). These results show that highly correlated gene pairs would disproportionately tend to share common regulators (the reverse however not being necessarily the case). A virtually identical result was obtained when these same analyses were conducted using alternative architectures with networks with either a fixed number of regulators of a normal distribution of regulators per target where a similar dependency for co-expression on co-regulation is observed (see S4 Fig). This finding suggests that the observed dependency between co-expression and co-regulation is robust to other network architectures.

**Fig 6:**
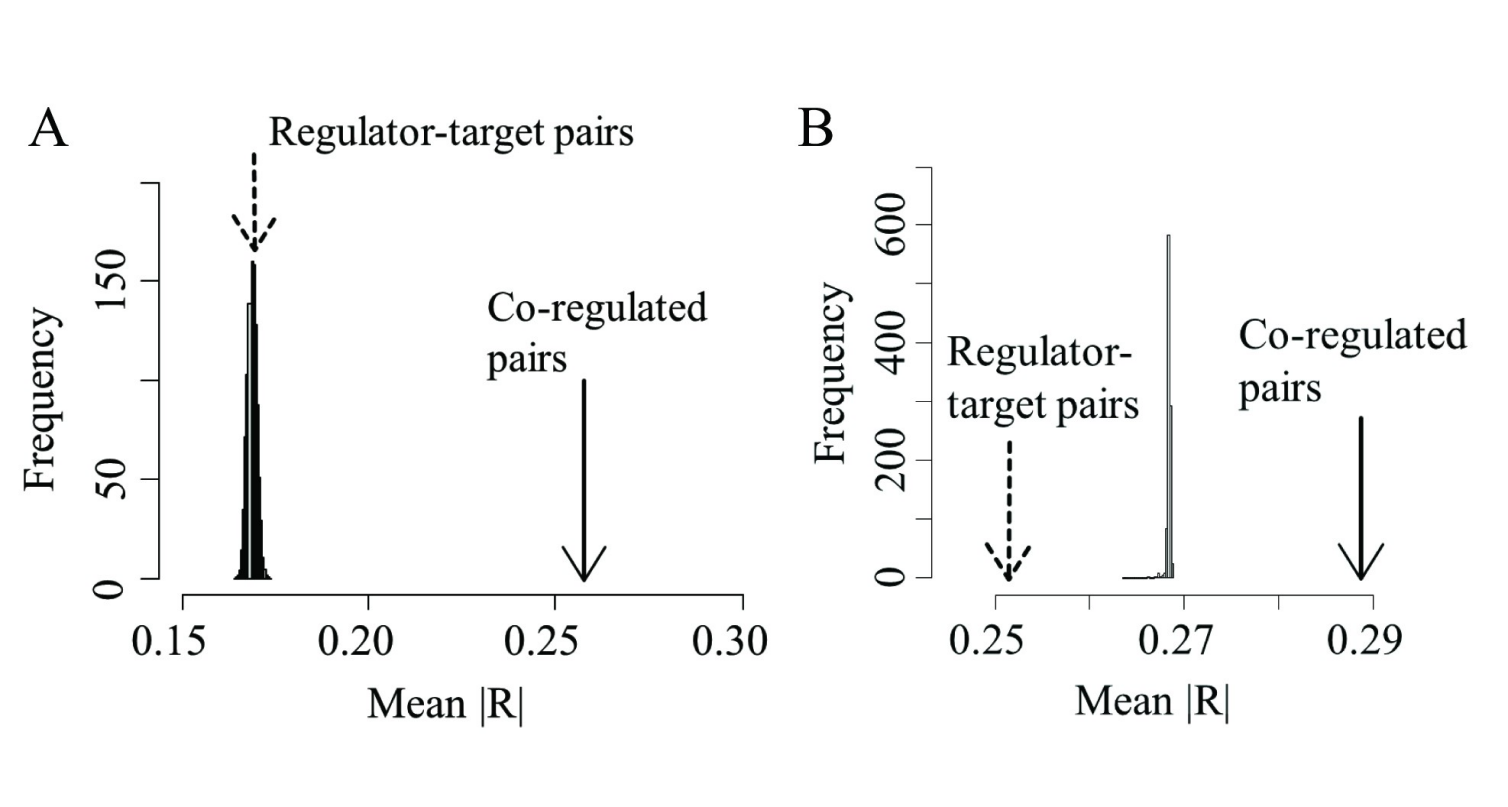
Pairs of genes sharing a common regulator tend to be more highly correlated than random background and regulator-target pairs of genes in both synthetic and natural transcriptomes. A) Average co-expression (absolute correlation) between those pairs of genes sharing a common regulator, as well as regulator-target pairsin the synthetic GRN (arrows) compared with the distribution of average correlations of 1000 equally sized samples of random background pairs of genes. B) Average coexpression (absolute correlation) between pairs of targets of either MYC, PAX2 or SRY, as well as regulator-target pairs involving all these three regulators, (pooled data, arrows) compared with the distribution of average correlations of 1000 equally-sized samples of random background pairs of genes using expression data derived from the Brainspan database.

In order to test this link between correlated expression and co-regulation, we reasoned that if over 90% of highly correlated gene pairs share common regulators, but not the other way around (that is, not all gene pairs sharing regulators will display correlated expression), we would expect that gene pairs known to be targeted by common regulators (shared TF binding sites), would tend to be, on average, more correlated with each other than background gene pairs. On the other hand, given the observed poor average correlation between regulators and targets, we would expect regulator-target pairs to display a much weaker average absolute correlation. We first tested this notion in our synthetic GRN simulations and measured the average absolute correlation of all existing gene pairs sharing at least one common regulator, as well as regulator-target pairs (n>15000 gene pairs, excluding genes with zero standard deviation, when occurring). We compared these two measures (see arrows in Fig 6A) with the distribution of average absolute correlations in 1000 equally-sized samples of gene pairs randomly drawn from the background gene population. We used random background gene pairs as a reference because in a real biological system it is currently impossible to know with certainty no transcriptional regulator is shared between any two given genes. Accordingly, we use background gene pairs as a reference point of comparison (rather than pairs of genes known not to share any transcriptional regulator). While this lack of complete knowledge is not the case for synthetic data, we chose to use the same reference to background gene pairs for consistency, and to make it directly comparable with the empirical expression data (next paragraph). As shown in Fig 6A, we found a highly significant bias, towards a higher average absolute correlation for gene pairs sharing common regulators. Regulator-target pairs, on the other hand showed an average absolute correlation no different to that of random background gene pairs.

Using brain expression data derived from the Brainspan dataset we measured the pooled average correlation between all possible pairs of known target genes for either MYC, PAX2 or SRY separately, as well as regulator-target pairs involving any of these three regulators, and compared them with the average correlation of 1000 equally-sized samples of random gene pairs drawn from the background gene population. As shown in Fig 6B, gene pairs jointly targeted by any of these transcription factors, displayed a significantly higher average absolute correlation (right arrow) than expected by chance (Fig 6B, histogram). Regulator-target pairs, on the other hand showed an average correlation significantly lower than random background gene pairs. This result confirms that, in line with our GRN simulations, gene pairs sharing regulators would tend to display a higher average correlation when compared with both background random gene pairs as well as with regulator-target pairs.

Taken together our results demonstrate that while immediate regulatory interactions between two genes (regulators-target pairs) fail to explain correlations in gene regulatory networks, co-expression emerges only when pairs of genes are being actively co-regulated.

## Discussion

In this study, we found that correlated patterns of gene expression observed in natural transcriptomes exceed the number of random correlations naturally expected by chance alone. This distinct structural signature has been hypothesized to reflect underlying regulatory and functional relationship between thousands of genes displaying coordinated levels of expression [13, 14, 17, 20, 21]. While functional relationships between co-expressed genes have been widely documented, [13, 14, 17–21], the exact nature of the regulatory relationships underlying correlated expression between groups of genes has remained poorly understood. Using simulations based on a synthetic GRN model we found that co-expression patterns naturally emerge under a wide range of regulatory architectures, and that these correlations also exceed simple random expectations. This clustered structure could potentially be the result of: A) a group of genes being under close regulatory proximity, in which case we could expect correlated genes to be in close proximity in the regulatory circuit. Or, B) a reduction in the total number of potential expression trajectories due to the global constraints imposed by the regulatory network, thereby forcing a large number of correlations even between genes located at any arbitrary distance in the regulatory network. In this study, we tested these two hypotheses by asking whether correlations occur between genes linked by the closest possible regulatory distance (regulator-target pairs), and found that the expression level of all targets is on average poorly or no correlated with that of their immediate regulators. Transcript levels are known to be poorly correlated with the level of the corresponding protein products, and, consequently, the expression level of a given transcription factor is not necessarily expected to be correlated with its regulatory activity [31]. However, at the transcriptome level the closest regulatory proximity between any two genes (regardless of any post-translational events) is the one that exists between a gene encoding for a transcriptional regulator and a gene directly targeted by it. So, in order to investigate the origin of correlated expression we needed to start by looking at the closest regulatory proximity between pairs of genes (at the transcriptome level). Even though our synthetic model does not take into account intermediate events between the expression of a transcription factor and its direct regulatory activity at the protein level, we still find a lack of correlation between the expression of regulators and targets. This result implies that, even when intermediate post-translational events are removed, co-expression still fails to emerge from the most direct regulatory interaction that exists between any two genes (regulator-target gene pairs). Instead, we find that high co-expression is to be found (both in simulated and real data), not between genes linked by the closest possible regulatory interaction (at the transcriptome level), but by pairs of genes further away in terms of regulatory proximity. Regarding regulator-target pairs, it is worth considering what would happen if time delays between regulator and target expression are considered in the analysis. This can be easily done using our synthetic model, by simply looking at the regulator’s expression at time t and corresponding targets at time t+1. As shown in S5 Fig of the Supporting material, a bimodal distribution emerges, revealing a large population of close-to-zero correlations and a smaller subpopulation of strong correlations (positive or negative depending on the regulator’s sign). It is however important to stress that obtaining this kind of data in actual experimental settings, currently constitutes a considerable technical challenges as it requires obtaining minutely detailed time series in synchronous cell populations. On the other hand, co-expression is currently documented in conditions in which expression data for both regulators and targets are obtained at the same time point, and is this observed co-expresssion the one we address in this study.

Given the lack of a direct correlation between the expression of a transcriptional regulator and its direct target, we asked whether highly correlated gene pairs could be located at any arbitrary regulatory distance within the control network (that is, no closer at a regulatory level than any two random genes). Using our GRN model, where the precise architecture of the transcriptional regulatory network is known, we measured the topological distance between every pair of highly correlated genes and found that highly coordinated genes were closer within the regulatory network than background genes and that, on average, any two highly correlated genes were linked by just one intermediary regulatory component. As this result strongly suggests that co-expressed genes share at least a single common immediate regulator, we measured the proportion of highly correlated gene pairs sharing a common immediate regulator compared with lowly correlated gene pairs and confirmed that over 90% of highly correlated gene pairs indeed shared immediate upstream regulators (the reverse, however, not being true). Our finding that the average correlation of all possible pairs of genes known to be targeted by any of three well-known transcription factors is significantly higher than the average correlation expected among random background gene pairs, further supports the notion that highly correlated gene pairs, in natural transcriptomes, share common immediate upstream regulators. The potential influence of other factors on co-expression, as normally measured in a range or experimental settings, has been recently examined in a study carried out by Farahbod and Pavlidis (2020). In it, the authors show that correlated patterns of expression in heterogeneous tissues (such as the brain tissue data used here), mainly reflect correlated changes in expression across cell types, as opposed to variations within cell types [32]. However, correlated changes in expression across cell types are also themselves a function of the underlying regulatory relationships and the focus of the present study is, precisely, the link between co-expression and the regulatory proximity of correlated genes given an underlying regulatory network. Our finding that genes sharing a common regulator tend to be more highly correlated than random background pairs of genes, even in heterogeneous brain tissue, lends further support to this notion. It is important to stress at this point that we use a synthetic gene regulatory network model to gain insights on possible sources of co-expression, and test these insights on empirical expression data. While a match between the insights gained with the synthetic model and the actual data suggests the underlying mechanisms could be similar, it does not unequivocally demonstrate that they are.

The assumption that co-expressed genes are more likely to share common regulators, has been examined in yeast where gene pairs with a very high expression correlation show a significant excess of shared binding sites in yeast [12, 17, 21]. However, in a subsequent study carried out in a more complex organism, Drosophila melanogaster, based on experimentally determined TFBS and microarray expression data, it was found that pairs of genes with shared TFBS show, on average, a higher degree of co-expression than those with no common TFBS [13]. However, the study in Drosophila also finds that gene pairs with high expression correlations do not share large numbers of TFBS [13]. In the latter study, the authors note that the microarray-based data used in their analysis is typically obtained from the whole organism and that the lack of relationship between the extent of co-expression and the number of shared TFBS may be related to the heterogeneity of cells and tissue types involved in these expression level data. It is worth noting that, in agreement with this possibility, the same authors find that when the same analysis is restricted to a short developmentally early time window, when the cell type heterogeneity is presumably less pronounced, a very large proportion of highly correlated gene pairs were found to share at least one TFBS [13]. Although the authors highlight that this relationship may be confined to a specific property of the early developmental programme, their result is consistent with our finding that the vast majority highly correlated genes will share common regulators. In this regard, our findings are also consistent with the relatively higher efficiency to infer regulatory sequences in mammalian genomes, when expression data from specific tissues are used, as opposed to using global expression data from the whole organism [13, 16, 33].

In summary, our results show that the levels of co-expression observed in simulated expression data are, just as in empirical data, significantly higher than expected by chance. When examining the source of correlated expression, we also find that high co-expression is unlikely to be the result of the closest existing regulatory proximity between any two genes at the transcriptome level (transcription factor-target gene relationships). Instead, highly correlated gene pairs tend to share at least one common regulator, while most gene pairs sharing common regulators do not necessarily display correlated expression. Our results also demonstrate that widespread co-expression naturally emerges in regulatory networks, and that it is likely to be a reliable and direct indicator of active co-regulation in a given cellular context.

## Supporting information

S1 Fig Synthetic GRNs generate realistic gene expression distributions. A) 3D plot showing the dependence of fluidity in the dynamic behaviour of the synthetic GRN model used in this study (percentage of frozen nodes or genes) as a function of a range of values for parameters A and B of the sigmoid function used to calculate the response of a target gene to a single regulator. B) Distribution of log-transformed gene expression data generated by the synthetic GRN model after 500 time points (iterations) in a typical network consisting of 1000 genes, with parameters A = 5 and B = 5, proportion of negative regulators = 0.4 and minimum number of regulators = 3. C and D) Distribution of log-transformed gene expression values in natural transcriptomes using RNA-seq based data from Brainspan and Fantom5 datasets respectively.
(TIF)

S2 Fig High co-expression in synthetic gene regulatory networks with alternative distributions of regulators per target. A) Co-expression clustering dendrogram based on expression data generated by a synthetic GRN after 1000 time steps (iterations), using a fixed number of regulators per target (n = 4 which corresponds to the mean number of regulators in the power law distribution used in Fig 2). B) The distribution of the absolute correlation of all synthetic gene pairs in the same network compared with the distribution resulting from random permutations of the same expression data. C) Co-expression clustering dendrogram based on expression data generated by a synthetic GRN after 1000 time steps (iterations), using a normal distribution of regulators per target (mean = 4, SD = 2, which corresponds to the mean and standard deviation of regulators in the power law distribution used in Fig 2). D) Distribution of the absolute correlation of all synthetic gene pairs for the above network compared with the distribution resulting from random permutations of the same expression data. Inset: Bars show the mean (±SEM) number of highly correlated pairs (/R/>0.5) obtained from 1000 independent GRN simulations compared with the expected mean number of highly correlated pairs (absolute correlation) resulting from random permutations of the gene expression values of the same networks.
(TIF)

S3 Fig Regulators and direct targets show poorly correlated expression levels in synthetic GRNs with fixed and normally distributed regulators per target. A) Distribution of correlation values of all individual regulator-target pairs involving only positive regulation in a network using a fixed number of regulators per target (n = 4, corresponding to the mean number of regulators in the power law distribution used in Fig 2). B) Distribution of correlation values of all individual regulator-target pairs from the same previous network involving only positive regulation. C) Distribution of correlation values of all individual regulator-target pairs involving only positive regulation in a network using a fixed number of regulators per target (mean = 4, SD = 2, which corresponds to the mean and standard deviation of regulators in the power law distribution used in Fig 2). D) Distribution of correlation values of all individual regulator-target pairs from the same previous network involving only negative regulatory interactions.
(TIF)

S4 Fig Highly correlated pairs of genes tend to share common regulators in synthetic GRN with alternative distributions of regulators per target. A) Bar chart showing the average proportion (±S.E.M) of pairs of genes sharing a common regulator among either highly correlated pairs (/R/ >0.8) or lowly correlated pairs (/R/ < 0.2) found in 1000 independent simulations using a fixed number of regulators per target (n = 4). B) Bar chart showing the average proportion (±S.E.M) of pairs of genes sharing a common regulator among either highly correlated pairs (/R/ >0.8) or lowly correlated pairs (/R/ < 0.2) found in 1000 independent simulations using a normally distributed number of regulators per target (mean = 4, SD = 2).
(TIF)

S5 Fig Effect of time delays on the observed correlated expression between regulators and targets. A and B) histograms showing the correlation distribution between either positive regulators and their targets (A) or negative regulators and their targets (B). C and D) histograms showing the correlation distribution between either positive regulators measured at time t, and their targets measured at time t+1 (C) or negative regulators at time t and their targets at time t+1 (D).
(TIF)

S1 File Matlab code for the synthetic GRN model. (M)

S2 File Example of simulated n = 1000 nodes GRN edge list. First column lists target nodes. Second column lists regulator and sign.
(TXT)

S3 File Example of simulated n = 1000 nodes GRN raw expression data. Running parameters: A = 5, B = 5, MinReg = 3.
(TXT)

S4 File (ZIP)

S5 File (ZIP)
