# Supplementary figures and images for: Emergence of co-expression in gene regulatory networks
Source: PLoS One. 2021 Apr 1;16(4):e0247671. doi: 10.1371/journal.pone.0247671 (PMC8016302; doi:10.1371/journal.pone.0247671)

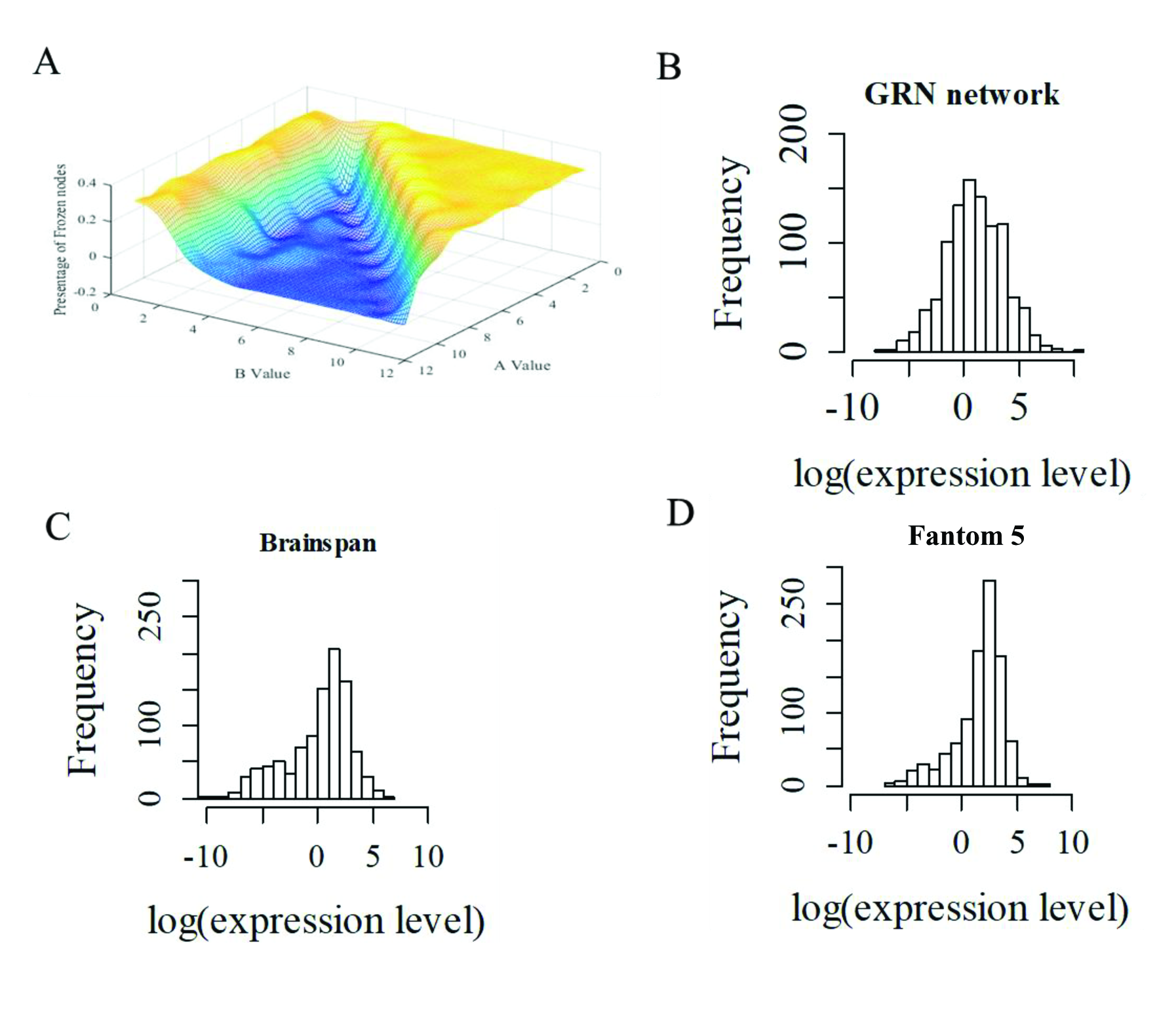

Supplement: S1 Fig — A) 3D plot showing the dependence of fluidity in the dynamic behaviour of the synthetic GRN model used in this study (percentage of frozen nodes or genes) as a function of a range of values for parameters A and B of the sigmoid function used to calculate the response of a target gene to a single regulator. B) Distribution of log-transformed gene expression data generated by the synthetic GRN model after 500 time points (iterations) in a typical network consisting of 1000 genes, with parameters A = 5 and B = 5, proportion of negative regulators = 0.4 and minimum number of regulators = 3. C and D) Distribution of log-transformed gene expression values in natural transcriptomes using RNA-seq based data from Brainspan and Fantom5 datasets respectively. (TIF) [file pone.0247671.s001.tif]

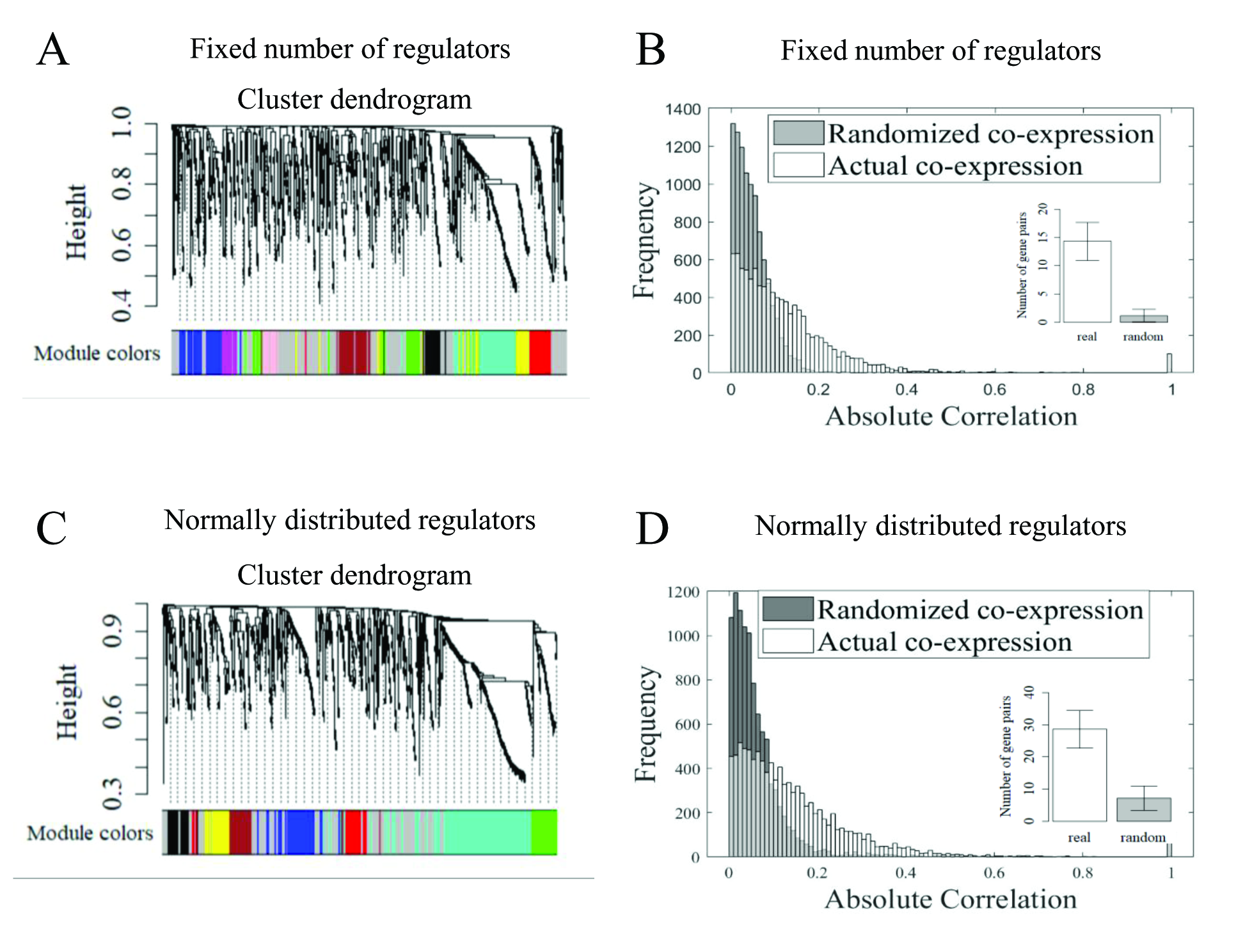

Supplement: S2 Fig — A) Co-expression clustering dendrogram based on expression data generated by a synthetic GRN after 1000 time steps (iterations), using a fixed number of regulators per target (n = 4 which corresponds to the mean number of regulators in the power law distribution used in Fig 2). B) The distribution of the absolute correlation of all synthetic gene pairs in the same network compared with the distribution resulting from random permutations of the same expression data. C) Co-expression clustering dendrogram based on expression data generated by a synthetic GRN after 1000 time steps (iterations), using a normal distribution of regulators per target (mean = 4, SD = 2, which corresponds to the mean and standard deviation of regulators in the power law distribution used in Fig 2). D) Distribution of the absolute correlation of all synthetic gene pairs for the above network compared with the distribution resulting from random permutations of the same expression data. Inset: Bars show the mean (±SEM) number of highly correlated pairs (/R/>0.5) obtained from 1000 independent GRN simulations compared with the expected mean number of highly correlated pairs (absolute correlation) resulting from random permutations of the gene expression values of the same networks. (TIF) [file pone.0247671.s002.tif]

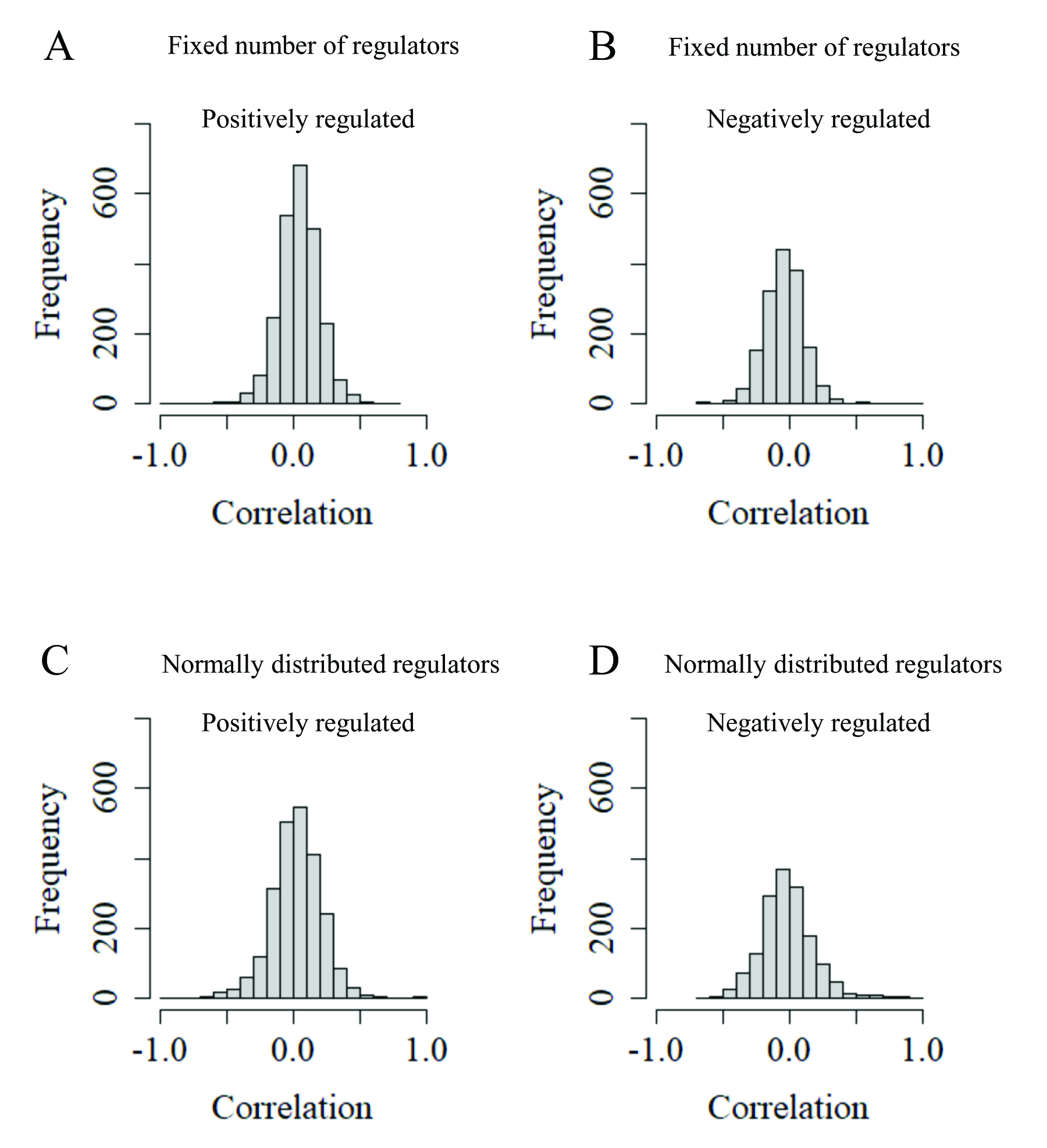

Supplement: S3 Fig — A) Distribution of correlation values of all individual regulator-target pairs involving only positive regulation in a network using a fixed number of regulators per target (n = 4, corresponding to the mean number of regulators in the power law distribution used in Fig 2). B) Distribution of correlation values of all individual regulator-target pairs from the same previous network involving only positive regulation. C) Distribution of correlation values of all individual regulator-target pairs involving only positive regulation in a network using a fixed number of regulators per target (mean = 4, SD = 2, which corresponds to the mean and standard deviation of regulators in the power law distribution used in Fig 2). D) Distribution of correlation values of all individual regulator-target pairs from the same previous network involving only negative regulatory interactions. (TIF) [file pone.0247671.s003.tif]

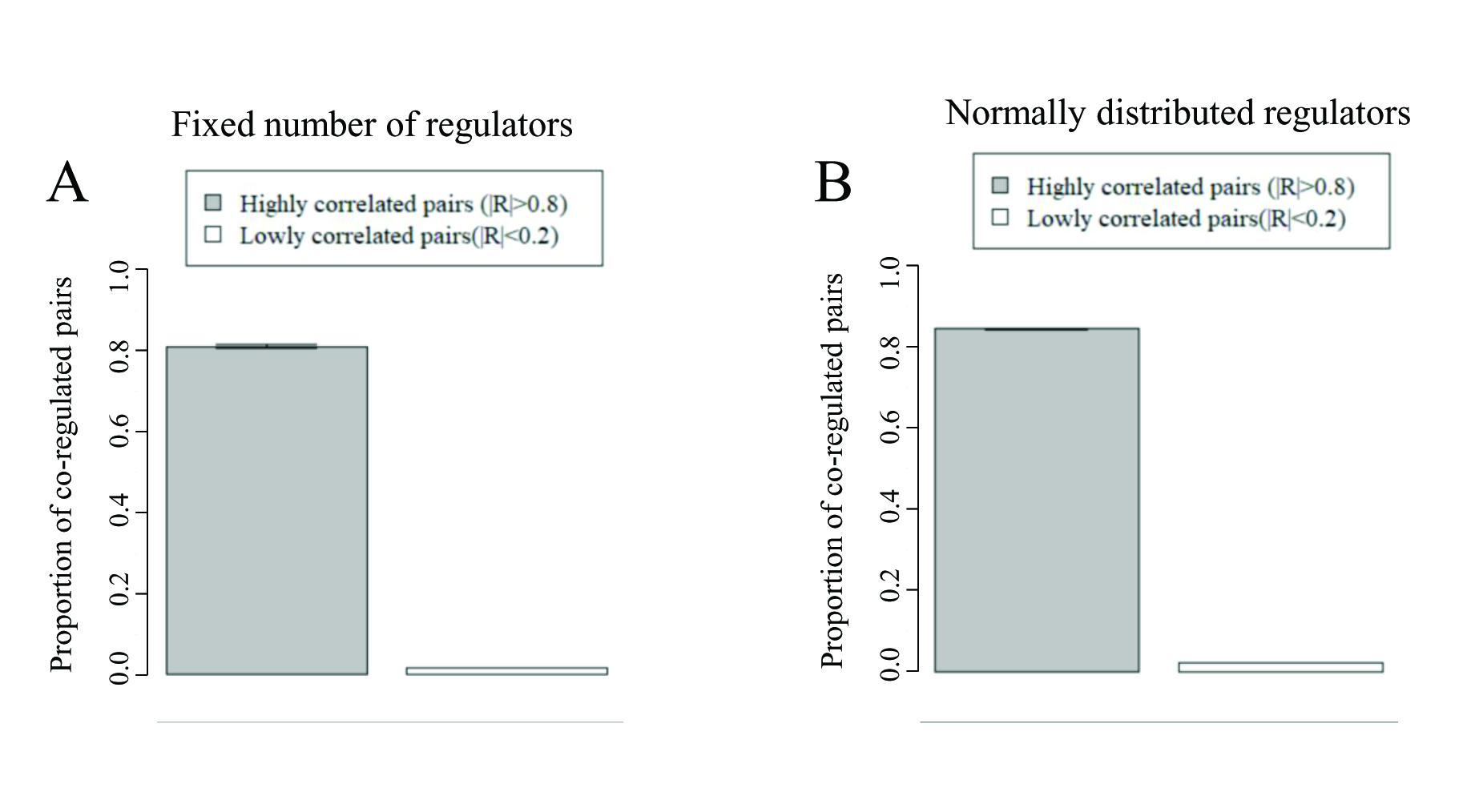

Supplement: S4 Fig — A) Bar chart showing the average proportion (±S.E.M) of pairs of genes sharing a common regulator among either highly correlated pairs (/R/ >0.8) or lowly correlated pairs (/R/ < 0.2) found in 1000 independent simulations using a fixed number of regulators per target (n = 4). B) Bar chart showing the average proportion (±S.E.M) of pairs of genes sharing a common regulator among either highly correlated pairs (/R/ >0.8) or lowly correlated pairs (/R/ < 0.2) found in 1000 independent simulations using a normally distributed number of regulators per target (mean = 4, SD = 2). (TIF) [file pone.0247671.s004.tif]

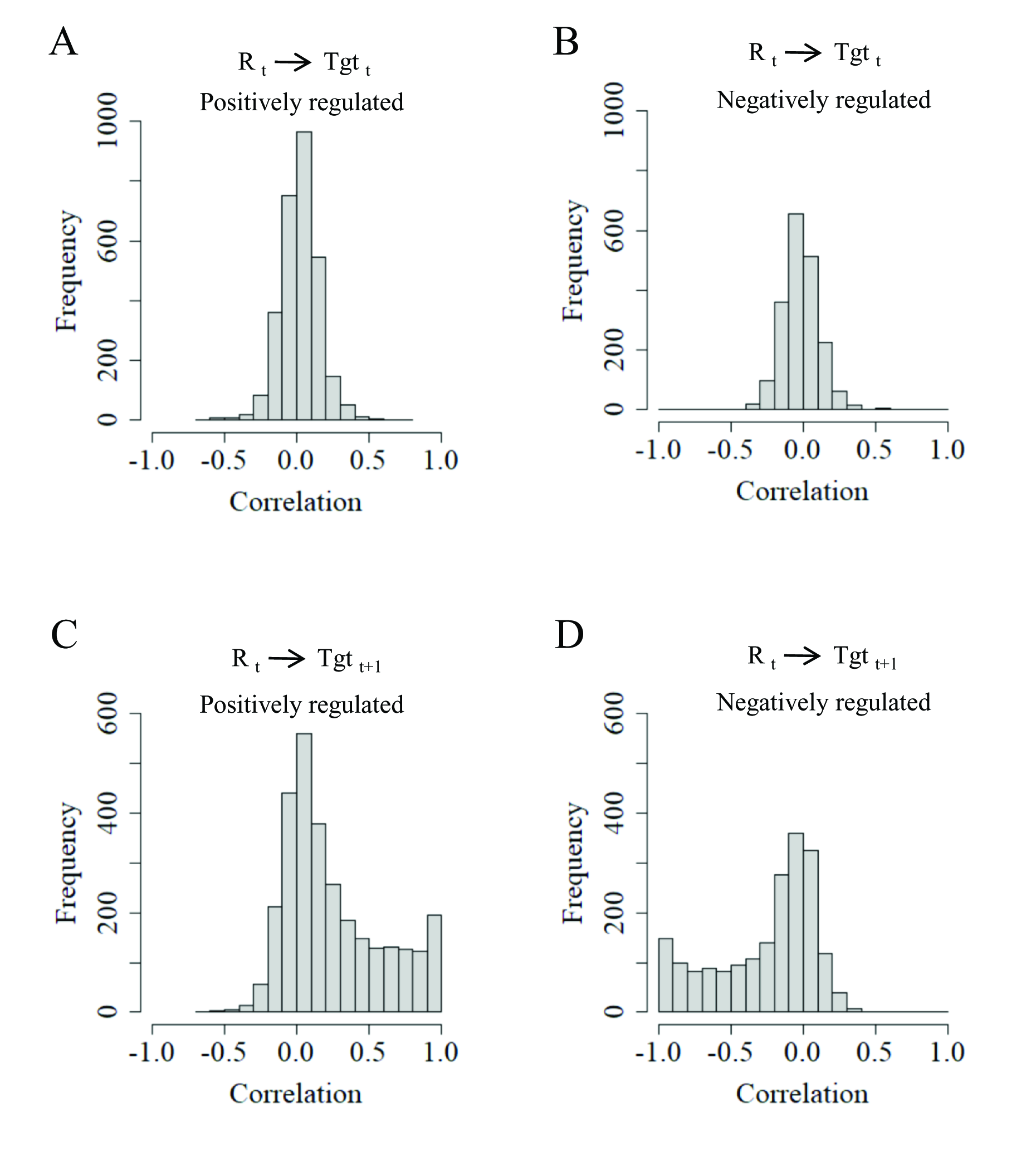

Supplement: S5 Fig — A and B) histograms showing the correlation distribution between either positive regulators and their targets (A) or negative regulators and their targets (B). C and D) histograms showing the correlation distribution between either positive regulators measured at time t, and their targets measured at time t+1 (C) or negative regulators at time t and their targets at time t+1 (D). (TIF) [file pone.0247671.s005.tif]
